# Memory Consolidation, Brain Atrophy, and Metabolism in Amyloid Positive and Negative Amnestic Mild Cognitive Impairment

**DOI:** 10.1002/alz.089852

**Published:** 2025-01-09

**Authors:** Camila Vieira de Ligo Teixeira, Cierra M Keith, Mark W Haut, Rashi Mehta, Holly Phelps, Melanie Ward, Mark Miller, R. Osvaldo Navia, Michelle M Coleman, Gary D Marano, Xiaofei Wang, Stephanie Pockl, Nafiisah Rajabalee, William T McCuddy, Pierre‐Francois D’Haese, Ali R Rezai, Kirk Wilhelmson

**Affiliations:** ^1^ Rockefeller Neuroscience Institute, West Virginia University, Morgantown, WV USA; ^2^ West Virginia University, Morgantown, WV USA; ^3^ St. Joseph Hospital and Medical Center, Phoenix, AZ USA

## Abstract

**Background:**

The existing literature has established that Alzheimer's disease (AD) is typically characterized by changes in memory‐associated temporal and parietal lobe atrophy and hypometabolism. However, some individuals clinically diagnosed with AD do not have biomarkers consistent with AD pathology. In this cross‐sectional study, we aimed to investigate differences in memory consolidation, temporal and parietal lobe atrophy, as well as temporal and parietal lobe metabolism within a clinically diagnosed cohort of individuals with amnestic Mild Cognitive Impairment (aMCI) who were either positive or negative for amyloid.

**Method:**

We retrospectively analyzed clinical data from 149 participants: 78 aMCI amyloid positive (A+), 48 aMCI amyloid negative (A‐), and 41 healthy subjects (HP). To analyze memory performance, we used the CVLT 1. % retained (CVLT%), 2. discrimination (CVLT‐D), 3. false positive (CVLT‐FP), and 4. intrusions errors (CVLT‐I). Entorhinal, supramarginal and angular cortical thickness were computed using CAT 12 and DKT atlas, and hippocampus volume using SLANT. A subset of the aMCI participants had an FDG‐PET, and we extracted the metabolism in the brain regions of interest for analysis. ANOVAs explored differences between groups on memory, brain atrophy, and metabolism (adjusted p<.002). Pearson’s correlations examined relationships between CVLT, MRI values, FDG‐PET measures, and biomarker data (adjusted p<0.0009).

**Result:**

A+ exhibited worse performance on CVLT%, CVLT‐D, and CVLT‐FP compared to A‐ and HP, and A‐ was worse on those tests compared to HP. There were no significant differences in the hippocampal volume or entorhinal thickness between biomarker status; A+ showed more atrophy in supramarginal and angular thickness than A‐ and HP. A+ showed hypometabolism in the entorhinal cortex compared to A‐. While hippocampus volume was related to CVLT%, CVLT‐D and CVLT‐FP, CVLT% was positively related to all the metabolism in all areas analyzed except hippocampus.

**Conclusion:**

In our memory clinic cohort characterized by biomarker status, our findings indicated that factors beyond AD might contribute to memory consolidation, temporal lobe atrophy, and temporal lobe. In addition, by inference, adding parietal atrophy to mesial temporal atrophy worsens memory consolidation.

